# Metformin May Contribute to Inter-individual Variability for Glycemic Responses to Exercise

**DOI:** 10.3389/fendo.2020.00519

**Published:** 2020-08-11

**Authors:** Steven K. Malin, Nathan R. Stewart

**Affiliations:** ^1^Department of Kinesiology, University of Virginia, Charlottesville, VA, United States; ^2^Division of Endocrinology and Metabolism, University of Virginia, Charlottesville, VA, United States; ^3^Robert M. Berne Cardiovascular Research Center, University of Virginia, Charlottesville, VA, United States

**Keywords:** pre-diabetes, type 2 diabetes, metabolic syndrome, insulin resistance, exercise, weight loss

## Abstract

Metformin and exercise independently improve glycemic control. Metformin traditionally is considered to reduce hepatic glucose production, while exercise training is thought to stimulate skeletal muscle glucose disposal. Collectively, combining treatments would lead to the anticipation for additive glucose regulatory effects. Herein, we discuss recent literature suggesting that metformin may inhibit, enhance or have no effect on exercise mediated benefits toward glucose regulation, with particular emphasis on insulin sensitivity. Importantly, we address issues surrounding the impact of metformin on exercise induced glycemic benefit across multiple insulin sensitive tissues (e.g., skeletal muscle, liver, adipose, vasculature, and the brain) in effort to illuminate potential sources of inter-individual glycemic variation. Therefore, the review identifies gaps in knowledge that require attention in order to optimize medical approaches that improve care of people with elevated blood glucose levels and are at risk of cardiovascular disease.

## Introduction

Nearly 34.2 million individuals in the U.S. have type 2 diabetes, and ~88 million men and women have prediabetes (1). Perhaps more concerningly is the observation that new cases of type 2 diabetes have increased significantly among U.S. youth, particularly non-Hispanic black people (1). This is clinically concerning because people with hyperglycemia are at greatly elevated risk for not only retinopathy, nephropathy, renal disease, but also cardiovascular disease (CVD). Blood glucose regulation is considered to be a complex balance between endogenous glucose production and peripheral glucose uptake. Insulin resistance of organs regulating these processes is considered to a be a primary defect. In particular, insulin resistance contributes to compensatory hyperinsulinemia via taxation on the pancreatic beta-cells to secrete insulin. Over time, however, the beta-cells begin to “fail” and cannot compensate for the ambient levels of systemic insulin resistance resulting in severe hyperglycemia. Therefore, targeting insulin resistance is a reasonable approach to the prevention, treatment, and management of type 2 diabetes.

**Figure 1:**
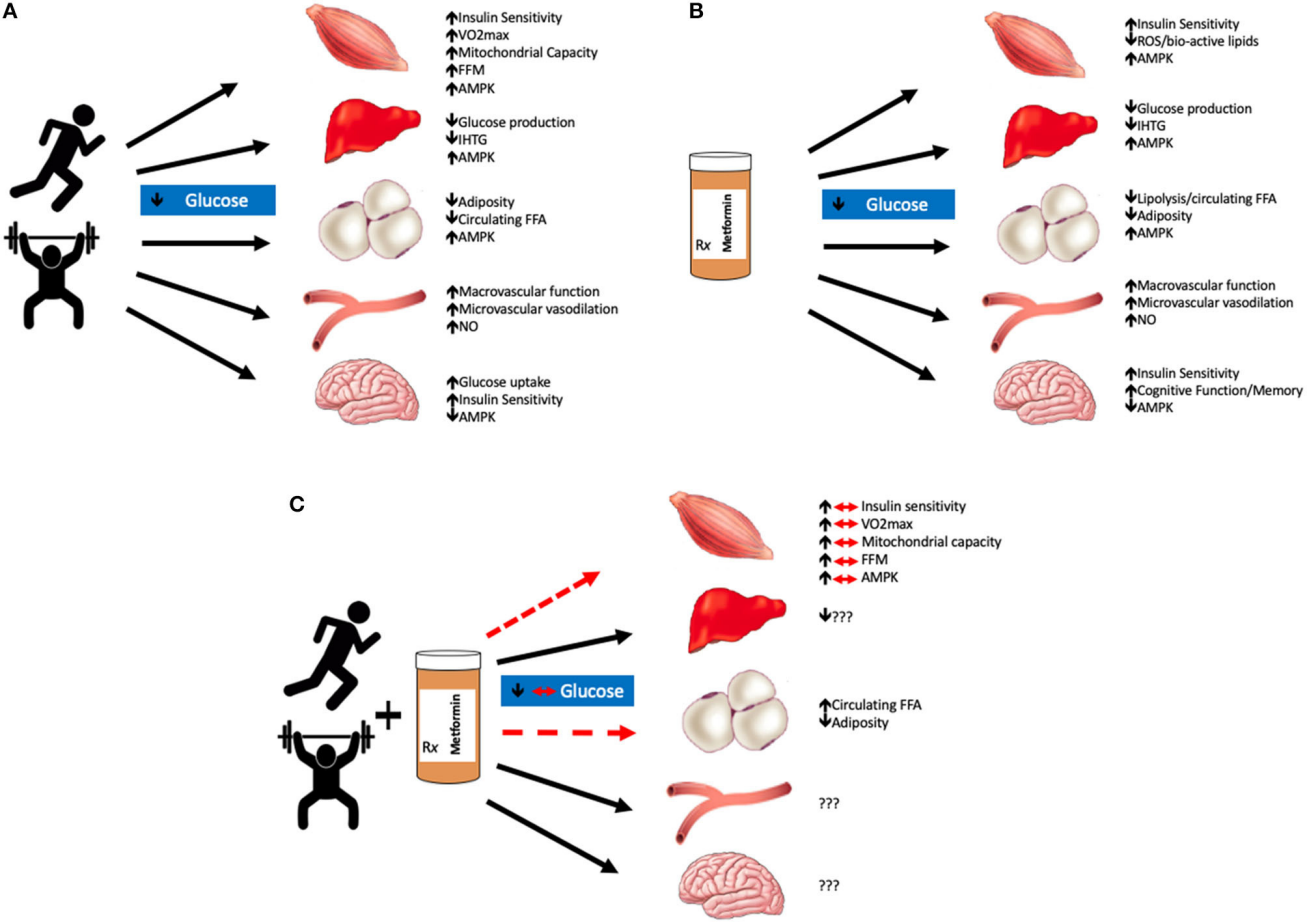
Summary of exercise and/or metformin interactions. Exercise lowers blood glucose mainly through increases in AMPK production in most organs excluding the brain, where production is decreased; and the vasculature, where adaptations are largely driven by nitric oxide (NO) **(A)**. Metformin alone also improves glycemic control through similar mechanisms, primarily by decreasing hepatic glucose production. Metformin also decreases reactive oxygen species (ROS) production which is suspected to improve tissue glycemic control as well as memory and cognitive function in the brain **(B)**. The combination of metformin with exercise has blunted effects on skeletal muscle glucose uptake and visceral adiposity. The effects of metformin with exercise on the liver, vasculature, and brain are still largely unknown **(C)**. We hypothesize that the combination of metformin and exercise are not necessarily additive in terms of glycemic control. Metformin blunts the beneficial adaptations that are typically seen with aerobic and/or resistance training in skeletal muscle tissue. VO2max, maximal oxygen consumption; FFM, fat-free mass; AMPK, adenosine monophosphate kinase; IHTG, intrahepatic triglycerides; FFA, free fatty acids; NO, nitic oxide.

Although randomized clinical trials show the efficacy of exercise to treat type 2 diabetes (2) as well as prevent the progression from prediabetes to type 2 diabetes (3, 4), there is large inter-individual heterogeneity in response to conventional exercise aerobic (up to 5 d/wk at 60–85% HR_max_) and strength (up to 2 d/wk at 60–80% 1-repetition max). Moreover, the optimal dose of exercise to improve glycemic control remains to be elucidated (5–7), and exercise adherence remains low. Patients with prediabetes and/or type 2 diabetes often exhibit multiple pathophysiological abnormalities that contribute to the approximate 20% lower aerobic capacity compared to those without dysglycemia (8). These include: mitochondrial dysfunction, poor muscle perfusion, and low cardiac function in addition to declines in pancreatic insulin secretion and sensitivity. Together, these are mechanisms contributing to decreased oxidative capacity and may help explain barriers to starting exercise interventions (9). Subsequently, many individuals may require pharmacological therapy to manage blood glucose concentrations. The American Diabetes Association suggests that in addition to lifestyle modification, metformin be considered the “first-line” pharmacological treatment to manage blood glucose in those with type 2 diabetes as well as those with prediabetes and at least 1 CVD risk factor (e.g., hypertension, elevated triacylglycerol, low HDL, etc.) (10). Not surprisingly, metformin is the most widely used prescription drug to treat hyperglycemia in adults with type 2 diabetes (11). In addition, metformin has gained interest in cancer prevention/treatment (12) as well as lifespan within aging (13). This highlights that metformin is a multi-faceted drug with health effects. Despite the widespread popularity of metformin, the interaction with exercise has received little attention. If anything, the overarching thought is that recommending exercise plus metformin will enhance glycemic control, and be better than either intervention alone. Herein, we highlight recent data describing whether co-prescribing metformin with exercise blunts, enhances, or has negligible effects on glucose regulation for ultimate CVD risk reduction. In this review, we focus on the multiple tissues (i.e., skeletal muscle, liver, adipose, vasculature, and brain) that metformin may affect during exercise training to influence cardiometabolic health (Figure 1). Lastly, we hypothesize that combining metformin with exercise may induce cellular processes that regulate metabolic adaptation in relation to glycemia.

## Impact of Metformin on Exercise Mediated Glycemic Control

Regulation of blood glucose is a tightly controlled process through “cross-talk” of pancreatic insulin secretion and insulin action on several tissues, including but not limited to: skeletal muscle, liver, adipose tissue, vasculature, and the brain. Fasting plasma glucose is maintained by endogenous (principally hepatic) glucose production, as glucose disposal by skeletal muscle and adipose tissue is minimal (14). Following mixed-meal absorption, insulin levels rise in response to carbohydrate (and to smaller extents protein) to reduce liver glucose production and lipolysis as well as stimulate blood flow to skeletal muscle for glucose uptake (15). Moreover, insulin acts on the brain to provide additional regulation of endogenous glucose production as well as inhibit additional food intake (16). Thus, considering treatments that impact liver and/or skeletal muscle glucose metabolism should, in theory, lead to enhanced glycemic control.

Current exercise prescription advised by the American College of Sports Medicine and the American Diabetes Association for men and women with prediabetes or T2D is to perform either 150 min/week or more of moderate intensity or 75 min/week of vigorous intensity aerobic exercise. The Look AHEAD study is a landmark clinical trial that reported a ≥ 7% reduction in weight loss through a nutrition intervention in combination with ≥175 min exercise/wk, reduced CVD risk by ~0.7%/yr (17), although more work is needed to understand the utility of lifestyle treatment for vasculature related events/mortality. Exercise can consist of either aerobic or resistance form, although the combination may result in the best HbA1c reductions (2). While there is still much debate as to whether exercise intensity is critical for glycemic control (18), we and others have shown either no effect (19) or that moderate intensity may have slightly better effects (20). Regardless, more recent work has suggested “exercise snacks” may be a novel approach to combat post-prandial hyperglycemia, and further work examining time of day to exercise is warranted (18).

Metformin predominantly reduces circulating glucose by lowering hepatic glucose production (11, 21), although it has also been reported to increase peripheral insulin sensitivity in some but not all work (22, 23). In the landmark U.S. diabetes prevention program, metformin was shown to decrease the incidence of T2D by 31% (1,700 mg/d) in adults with impaired glucose compared with 58% following lifestyle modification (7% weight loss and 150 min/wk of physical activity) (4). Although these findings suggest lifestyle was better than metformin alone, the Indian Diabetes Prevention Program (IDDP) observed that both regular physical activity (recommended >30 min/d) and metformin (500 mg/d) reduced the progression of impaired glucose tolerance to T2D in native Asian Indians (24).

Exercise and metformin both increase 5-adenosine monophosphate kinase (AMPK). This is important because AMPK is one of the several mechanisms by which each therapy act to suppress hepatic glucose output and increase insulin-stimulated glucose disposal (11, 23). As a result, it would be fair to expect a greater benefit to glycemic control since two of the major organs regulating blood glucose would be impacted compared with either treatment alone. However, the literature on co-prescribing lifestyle modification with metformin on blood glucose is equivocal (Table 1). Indeed, some (25) have shown that lifestyle modification plus metformin resulted in more weight loss than lifestyle modification alone, and the weight loss was associated with lower 2-h circulating glucose levels. This is somewhat consistent with recent work by Erickson et al. (26) showing that post-meal exercise and metformin resulted in the lowest peak post-prandial glucose excursion compared with either treatment alone in people with hyperglycemia. Furthermore, Ortega et al. sought to test the effects of combining metformin with exercise on free-living glycemic control in individuals with prediabetes or T2D (27). The results of this later work demonstrated that high intensity interval exercise in combination with metformin therapy lowered interstitial fluid glucose to a greater extent than exercise alone. Interestingly, others have suggested that in people with T2D treated with metformin that timing exercise 30 to 60 min following drug ingestion may impact plasma glucose and insulin to a greater extent than exercising 90 min after ingestion (28). The Diabetes Aerobic and Resistance Exercise (DARE) trial, however, showed that people with T2D on metformin plus lifestyle modification had similar HbA1c improvements when compared with individuals on lifestyle modification only (29). This is consistent with the IDDP since it was shown that the combination therapy of metformin and lifestyle modification had equivalent effects to reduce the progression from prediabetes to T2D (24). Notwithstanding this, a retrospective analysis of the Look AHEAD study demonstrated that people with type 2 diabetes treated with metformin prior to and during intensive lifestyle therapy had smaller improvements in fasting plasma glucose and HbA1c compared with those undergoing lifestyle therapy only (30). Further, the work by Boulé et al. (31) tested the effect of metformin on glycemic control in response to a single bout of submaximal aerobic exercise at ~33, 67, and 79% of VO_2_peak and resistance exercise (i.e., leg extension and flexion) in individuals with T2D. Their results implied that metformin blunted reductions in post-prandial blood glucose concentrations during a standardized meal. Together, most (26, 27) but not all (24, 31) studies showing an additive effect of metformin plus exercise studied individuals who were already prescribed metformin. In contrast, we showed previously (32) that 12 weeks of metformin plus exercise training prospectively in naïve users had no effect on fasting plasma glucose in adults with prediabetes. This is consistent with newer work (33, 34) whereby in normoglycemic insulin-resistant adults, fasting or postprandial glucose levels did not appear to be negatively affected by metformin. Somewhat surprisingly, however, is the observation that no randomized clinical trial has been designed to date to test the effectiveness of exercise plus metformin on glycemic control. Given that some work shows opposing (31), additive (26, 27), or null findings (32–34), it is reasonable to suggest that metformin contributes to inter-individual glycemic response differences (Table 1).

**Table 1:** Summary of clinical trials examining the impact of metformin in combination with exercise on glycemic control compared to exercise alone.

**Study details**	**Prescription/Population**	**FPG**	**2-h Postprandial**	**HbA1c**
Ramachandran et al. (24)	Walk or cycle >30 min/d + 1,000 mg/d metformin for ~3 years in overweight/obese adults	↔	↔ during OGTT	N/A
Sharoff et al. (23)	An acute bout of cycle ergometry for 30 min @ 65% VO_2_Peak + 2–3 weeks of 2,000 mg/d metformin treatment prior in overweight/obese adults	↔	N/A	N/A
Love-Osborne et al. (25)	Self-chosen life style change + 1,700 mg/d metformin for 6 months in overweight/obese adolescents	↔	↔ during OGTT	N/A
Erickson et al. (26)	Postmeal exercise (5 x 10-min bouts of treadmill walking at 60% VO_2_Peak) + 1,000–2,000 mg/d metformin in overweight/obese adults	N/A	↓ during MMTT	N/A
Ortega et al. (27)	An acute bout of exercise + physician prescribed dose of metformin in insulin-resistant adults	N/A	↔ during OGTT	N/A
Boulé et al. (31)	~33 submaximal exercise bouts lasting 3–15 min @ 67–79% VO_2_Peak + 28 d of 2,000 mg/d metformin treatment prior in adults with T2D.	↑	↑ during MMTT	N/A
Terada and Boulé (30)	Nutrition intervention/with ≥175 min exercise/wk + metformin therapy in overweight adults with T2D	↑	N/A	↑
Malin et al. (32)	60–75 min of moderate-high intensity concurrent training 3 d/wk + of 2,000 mg/d metformin administration for 12 weeks in adults with prediabetes	↔	N/A	N/A
Konopka et al. (33)	45 min of moderate-high intensity cycle ergometry 3 d/wk + 2,000 mg/d metformin for 12 weeks in older adults with prediabetes	↔	N/A	↔
Walton et al. (34)	PRT + 1,700 mg/d metformin for 14 weeks in healthy older adults	↔	N/A	N/A

*FPG, fasting plasma glucose; OGTT, oral glucose tolerance test; MMTT, mixed meal tolerance test; N/A, Not applicable because the measurement was either not reported or measured; ↑, higher; ↓, lower; ↔, no difference*.

## Effect of Metformin on Exercise-Mediated Skeletal Muscle Insulin Sensitizing Effects

Exercise improves glycemic control through both skeletal muscle insulin-dependent and insulin-independent mechanisms (35). Subsequently, contraction mediated mechanisms favoring glucose uptake last for ~3–6 h following a single bout of exercise. In time, insulin-sensitizing effects take over to explain improved glucose control (36). Habitual exercise (i.e., lifestyle change) is recommended to reduce T2D risk in part by maintaining skeletal muscle glucose disposal.

Metformin is suggested to stimulate skeletal muscle glucose uptake and oxidation (37). Moreover, metformin has been shown to lower intramuscular triglyceride content and bioactive acyl-chain bioactive lipids (38, 39) through in part elevations in fat oxidation. Together, these observations indicate that metformin has effects on skeletal muscle energy metabolism that favor glucose homeostasis.

Because metformin is advised as a first-line pharmacological agent, we conducted a double-blind, randomized control trial to test the effect of exercise training with and without metformin on insulin sensitivity in people with prediabetes (32). For 12 weeks, individuals were randomized to either: placebo, metformin, exercise training with placebo, or exercise training with metformin. All people were provided metformin at 2,000 mg/d or a placebo, while those randomized to exercise underwent a progressive aerobic and resistance training program at 70% of their individual heart rate peak and 1-repetition max, respectively. Insulin sensitivity was determined about 28 h post-exercise via the euglycemic-hyperinsulinemic clamp with glucose isotope tracers. Tracers were utilized to determine the effects of metformin on skeletal muscle insulin sensitivity as well as hepatic glucose production. The primary results showed that metformin blunted exercise mediated increases in insulin-stimulated skeletal muscle glucose uptake by ~30%, suggesting that metformin diminishes both single and repeated bouts of exercise benefit on glucose metabolism (23, 31, 32). Although to date no follow up studies have been conducted using stable isotopes to understand skeletal muscle insulin-stimulated glucose disposal, recent work has tested the effect of metformin on aerobic or resistance exercise skeletal muscle cellular adaptation (33, 34). The results of these studies collectively show that metformin opposes skeletal muscle mitochondrial adaptations as well as inhibits fat-free mass accretion (see below *Cell Mechanisms* for further discussion), which were directly correlated with attenuated gains in aerobic fitness as well as strength. Together, these findings highlight that blunted fitness adaptation may relate to the reduced skeletal muscle insulin sensitivity response. In either case, this smaller gain in insulin sensitivity following the combination of exercise and metformin treatment does not apparently lead to stark blood glucose elevations (23, 31, 32). Further work is warranted to better understand how the combination of drug-exercise therapies contributes to glycemic control across exercise doses, particularly in people with T2D. For instance, recent work demonstrated that metformin increased carbohydrate utilization during high intensity interval exercise in insulin resistant adults when compared to exercise alone (27). This may be of clinical relevance since carbohydrate use during exercise was related to insulin sensitivity as measured by the intravenous glucose tolerance test. The findings of Ortega et al. (27) also suggest that exercise intensity may interact with metformin to positively influence insulin-stimulated glucose uptake when compared with moderate intensity exercise (31, 40). Whether exercise intensity interacts with metformin to affect skeletal muscle insulin sensitivity in clinical populations remains to be tested to help understand if muscle is the primary driver of glycemic variation responses.

## Effect of Metformin on Exercise-Mediated Liver Insulin Sensitizing Effects

Hepatic glucose production results from gluconeogenesis and/or glycogenolysis, and people with impaired fasting glucose display elevated hepatic glucose production (25, 41), or inappropriately normal levels given the prevailing hyperinsulinemia (40). Indeed, people at risk for or with T2D, in particular, have impaired responses to insulin (42). This highlights that the liver becomes insulin resistant and plays roles in both fasting and fed states. While fasting glucose (and insulin) may serve as a proxy for hepatic glucose production, and study of hepatokines, liver fat, or liver enzymes (43) may provide indirect estimates of hepatic function, use of stable isotopes along with hyperinsulinemic-clamps represent ideal methodologies to depict the role of the liver on glycemic control.

The exercise impact on hepatic glucose production is generally positive. One to seven days of aerobic exercise has been shown in people with T2D to increase hepatic insulin sensitivity (44, 45). Exercise training studies of ~12 weeks have also demonstrated favorable effects on hepatic insulin sensitivity (46), with at least some of the effect being related to improved hepatokines (i.e., fetuin-A) (46). However, it is worth noting that others have suggested that re-feeding calories expended from exercise negates these liver insulin-sensitizing benefits of exercise in adults with excess weight/insulin resistance (47). It cannot be ruled out though that discrepancies between short-term training studies may relate to exercise intensity, as higher intensity exercise activates AMPK in hepatocytes (48). As a result, it seems that energy deficit, at least partially, created by exercise is an important mechanism improving hepatic insulin sensitivity.

Metformin improves hepatic insulin sensitivity. The mechanism by which metformin lowers hepatic glucose production is mainly thought to be through activation of AMPK and reduction in gluconeogenic enzymes (49), although some suggest antagonism of glucagon may be important (50). In addition, metformin is considered to increase fat oxidation in hepatocytes, thereby reducing the potential delirious effects of lipids on insulin signaling (51). Recent work has suggested that metformin may benefit conditions of hepatic steatosis. In particular, although metformin-induced similar reductions in the hepatic triglyceride content of Otsuka Long-Evans Tokushima Fatty (OLETF) rats under caloric restriction, compared to caloric restriction alone, the combined treatment lowered hepatic-derived inflammation more (52). Additionally, metformin augmented the benefits of caloric restriction on lowering post-prandial circulating glucose in rodents, suggesting that metformin may impact the liver during energy deficit reduce diabetes and non-alcoholic fatty liver disease risk (52). This observation of greater glycemic benefit was in parallel to greater beta-oxidation and mitochondrial mitophagy (i.e., BNIP3).

To date, we are aware of only one study in humans that has systematically tested the effect of combining metformin with exercise on hepatic glucose production (32). In this study, we showed that 12 weeks of metformin, exercise, or the combination of therapies maintained hepatic glucose production as measured by stable isotopes despite reductions in fasting plasma insulin. This highlights that all treatments improved hepatic insulin sensitivity in middle-aged adults with prediabetes. Thus, it would seem the liver is unlikely to explain glycemic variation post-exercise. Further work in humans is required to understand, nevertheless, how exercise and metformin interact to affect hepatic function given that fatty liver disease is prominent in people with obesity and T2D, and fatty liver disease plays a critical role in the development of CVD.

## Influence of Metformin and Exercise Adipose Tissue Insulin Action

Adipose tissue is the primary supplier of plasma free fatty acids (FFA). FFAs provide energy to working tissues, including skeletal muscle and liver primarily during fasting states. In response to mixed meals (i.e., carbohydrate, protein, and fat), insulin suppresses lipolysis due to a feedback loop with the pancreas (53), and lowers circulating FFA to enable insulin action on the peripheral for glycemic control. However, when adipose tissue becomes resistant to the action of insulin, FFA concentrations rise in circulation and play an important role in the development of insulin resistance (54). In fact, the release of FFAs from adipose tissue contributes, not only to declines in skeletal muscle and hepatic insulin sensitivity but also to endothelial dysfunction and reduced β-cell function in obesity, prediabetes, and T2D (55–57). The reason FFAs contribute to this multi-tissue insulin resistance is beyond the scope of this review, but likely relates to elevated plasma FFA concentrations being linked with reduced mitochondrial function and metabolic flexibility (58), Therefore, it would be reasonable to expect aerobic exercise interventions designed to improve oxidative capacity to not only protect against FFA-induced insulin resistance but also improve adipose insulin action.

Exercise confers several benefits to adipose tissue that include reductions in not only total fat mass but also visceral adiposity (59). A consequence of this improved body fat mass has been proposed to decrease circulating FFAs as well as inflammatory mediators referred to as adipokines. Indeed, we have shown that changes in circulating FFAs following moderate intensity training are directly related to improved peripheral insulin sensitivity (32) and short-term interval or continuous exercise increases adipose insulin sensitivity in adults with prediabetes (19). While reductions in body fat following exercise training may be a key explanation for reducing circulating FFAs (60) in relation to improved peripheral insulin sensitivity and CVD risk reduction, fat loss is not required for improved adipocyte function. In fact, we recently showed that energy deficit, but not fat mass reduction, is important for improving adipokine profiles during caloric restriction (61). Moreover, Heiston et al. demonstrated that just 2 weeks of aerobic interval or continuous exercise increased adiponectin and lowered leptin prior to clinically meaningful weight loss or reductions in fat mass in older adults with prediabetes (62). Regardless, prior work (63) showed that hepatic insulin sensitivity was increased more following exercise training with a hypocaloric diet than when compared with a eucaloric diet during lipid-infusion. This suggests that in addition to exercise, calorie restriction may protect the liver from obesity-driven insulin resistance more so than training alone, despite comparable peripheral insulin sensitivity (64). Taken together, exercise, with or without caloric restriction, is an effective treatment for improving adipose tissue function.

Metformin is known to induce weight loss in adults with obesity, prediabetes, and T2D (65). Metformin reduces circulating FFA in part through inhibiting lipolysis (66). In fact, in murine adipocytes, metformin activated AMPK and blunted ANP as well as catecholamine-stimulated lipolysis (67, 68). Interestingly, elevated and/or blunted reductions in circulating FFAs have been reported after metformin plus exercise treatment during rest, exercise, or insulin-stimulated conditions compared to exercise alone (23, 31, 32, 69). While recent work suggests that oral metformin administration does not impact subcutaneous adipose tissue lipolysis during submaximal exercise in young lean men (70), it remains possible that in clinical populations alterations in either adipose lipolysis or reduced clearance as well as esterification may contribute to higher plasma FFAs. In either case, the elevated FFAs have been correlated attenuated gains in insulin sensitivity following metformin plus exercise therapy (23, 32). This may be clinically important as intrahepatic fat accumulation was lowered more after a diet and exercise than when lifestyle therapy was combined with metformin in obese adolescents (71). The blunted improvement in hepatic steatosis in these adolescents is consistent with the view that elevated FFAs from adipose tissue travel through the portal vein to the liver for increasing hepatic lipid storage. Collectively, this work highlights that adipose-derived metabolism may play a role in CVD risk following the co-prescription of metformin and exercise.

## Influence of Metformin and Exercise Vasculature Function

Insulin promotes vasodilation in large conduit arteries and resistance arterioles as well as microvasculature perfusion (72). Conduit and resistance arteries are important for the delivery of nutrients and oxygen to metabolically active tissues, whereas the microvasculature provides a critical role in the exchange of these substances. In turn, adequate insulin-stimulated blood flow and endothelial function are essential for glucose regulation. However, during periods of physical inactivity and/or nutrient excess, hyperinsulinemia develops and has been related to elevated endothelin-1 (ET-1) mediated vasoconstriction. This impaired glucose delivery may not only increase risk for T2D but also contribute to endothelial dysfunction through lower nitric oxide bioavailability. Interestingly, people with insulin resistance have been noted to have normal fasting vascular function, but impaired conduit or microvascular insulin action (73). This demonstrates that mechanisms underlying disease states may be unique in the fasted vs. insulin-stimulated state.

Habitual physical activity elevated insulin-mediated skeletal muscle glucose disposal and limb blood flow (65, 74). The dose at which exercise impacts vascular insulin sensitivity, however, is less clear. Although recent work suggests that interval exercise improves flow-mediated dilation (FMD), which measures large conduit arteries, more than continuous exercise in sedentary people (11, 12, 75) not all studies agree (76). Interestingly, we recently studied the effect of interval vs. continuous exercise on fasting and post-prandial arterial stiffness as well as endothelial function as measured by FMD in older adults with prediabetes (77, 78). We found that 2 weeks of high intensity interval or moderate continuous exercise reduced post-prandial arterial stiffness but had no overall effect on fasting or post-prandial FMD. Nonetheless, when examination of responder compared with non-responder analysis was performed, it was shown that continuous exercise elicited a 57% response rate to raise FMD compared with only 42% with interval exercise (78). This latter finding is consistent with work showing that either a single bout or short-term exercise training at moderate continuous intensity can promote vasodilation after glucose-induced insulin stimulation in adults with and without T2D (79–82). Therefore, exercise appears to exert unique effects on the vasculature in fasted compared with fed (or insulin-stimulated) states based on the intensity at which exercise is performed in clinical populations. While these studies tested vascular function under a glucose load, no study to date has investigated the effect of lipid infusion on endothelial function before or during insulin-stimulation following training. However, aerobic fitness has been directly correlated with the preservation of insulin-stimulated microcirculatory function in healthy young adults (83). Moreover, in healthy inactive young adults, 12 weeks of interval exercise was shown to increase brachial artery conduit artery function more so than continuous training alone during a high fat meal (84). Together, fitness mediated mechanisms may be important for opposing FFA-induced vs. glucose-induced skeletal muscle vascular insulin resistance.

Metformin improves brachial artery FMD in people with type 1 diabetes (85) and polycystic ovarian syndrome (86). Moreover, metformin treatment for 4 weeks increases forearm blood flow and glucose uptake following a 75 g glucose load in people with T2D (87). Interestingly, this improvement in forearm blood flow corresponded with improved glucose tolerance and lower FFA levels, suggesting lower gluco-lipid toxicity may contribute to improved endothelial function. Given that insulin-mediated glucose uptake is more closely associated with microvascular blood flow than total flow (88), it is important to understand the role of metformin on microvasculature function. To date, no data exist in humans studying the impact of metformin on microcirculatory function. Recently, Bradley et al. though showed that 2 weeks of metformin treatment improved microvascular responses during a euglycemic-hyperinsulinemic clamp in the muscle of high-fat fed rat (89). In particular, metformin lowered body weight and FFAs as well as improved insulin-stimulated muscle Akt phosphorylation, which confirms improved insulin signaling. Although there was no change in muscle AMPK phosphorylation, these findings suggest that metformin impacts nutrient exchange with skeletal muscle for glucose uptake. This is consistent with the notion that metformin increases eNOS phosphorylation in cultured endothelial cells (90). While work in human microvasculature insulin sensitivity awaits further investigation, metformin appears to have a direct effect on vasculature insulin action in skeletal muscle.

Traditionally, chronic exercise reduces CVD risk by decreasing blood pressure, triglycerides (TG), and inflammation (91). Metformin is not only used to treat T2D but also it is suggested to lower CVD risk (11). Indeed, the UK Prospective Diabetes Study (UKPDS) was a multi-center trial demonstrating that using pharmacological agents like metformin reduced HbA1c by ~11% over 10 years as well as lowered microvasculature endpoints (e.g., retinal photocoagulation) (10, 92, 93). However, there are few data from randomized trials examining if metformin alters the vasculature adaptation to exercise. From our observations of blunted insulin sensitivity following the combined treatment (32), we studied the impact metformin would have on exercise-mediated improvements in CVD risk factors (i.e., blood pressure, inflammation, and blood lipids) (94). Interestingly, metformin or exercise training monotherapies lowered systolic blood pressure and C-reactive protein (CRP) by ~7–8 and 20–25%, respectively, in people with prediabetes. When metformin and exercise were combined though, blunted reductions in systolic blood pressure and CRP were observed. These data were in line with others reporting that combining metformin with a low-fat diet and increase physical activity program had no further improvement in blood pressure (54). Furthermore, our observations were confirmed in obese insulin resistant adolescents whereby the metformin plus lifestyle modification blunted reductions in CRP as well as fibrinogen (71). Taken together, the metformin plus exercise therapy has strong clinical potential to oppose the reversal of chronic disease, including hypertension and metabolic syndrome. Further work is required for elucidating the vascularture mechanism(s) (e.g., FMD or angiogenesis) by which metformin interacts with exercise to lower or prevent CVD risk in people at risk for T2D.

## Metformin and Exercise on Brain Insulin Sensitivity

Insulin impacts the central nervous system by regulating hepatic glucose production, food intake and adipose metabolism, vasodilation/vasoconstriction of blood vessels as well as pancreatic insulin secretion and skeletal muscle insulin sensitivity (95, 96). Although these effects of insulin are clearly important for systemic glucose control, more recent work highlights that insulin also impacts memory, mood, and cognition (97, 98). Interestingly, Williams et al. (99) demonstrated direct effects of insulin on memory using intravenous insulin administration via a hyperinsulinemic-euglycemic clamp in 12 healthy older adults. In particular, this improvement in memory was related to increased blood oxygen level-dependent (BOLD) signaling as measured by functional MRI (fMRI) during the clamp (99). Furthermore, improved memory was best in those individuals with the highest systemic insulin sensitivity. This suggests that declines in insulin sensitivity may contribute to brain pathology in the hypothalamus (95). Not surprisingly, this may relate to cognitive decline (100), cerebral atrophy (101) as well as low brain blood flow and metabolism across aging (102). Additionally, this altered brain insulin action may be a key pathological factor in regulating glycemic control in individuals with obesity, T2D, aging, and Alzheimer's disease (103, 104).

During exercise brain glucose uptake declines in an intensity-based manner (105). This is likely the result of increased substrate availability (e.g., lactate) that allows glucose to be used by other tissues, such as skeletal muscle and red blood cells, for energy production. Conversely, aerobic interval exercise (4 x 4 min > 90% VO_2_peak) for 3 d/wk combined with moderate intensity exercise (70% VO_2_peak) for 2d/wk training has been demonstrated to increase basal glucose uptake in brain regions critical to cognitive function in young and older adults (106). Interestingly, the latter findings were observed in the parietal-temporal and caudate regions, which are linked to Alzheimer's disease. In either case, there remains limited data in humans with obesity or T2D confirming the effects of exercise on brain insulin sensitivity in relation to glucose metabolism. It was shown that lifestyle modification inducing weight loss, including increased physical activity and low-fat diet, increased brain insulin sensitivity in people with obesity as assessed by intranasal insulin spray (107). Moreover, Honkala et al. (108) demonstrated in sedentary middle-aged adults with insulin resistance sprint interval training for 2 weeks lowered insulin-stimulated glucose uptake in the temporal cortex, cingulate gyrus, cerebellum as well as global regions when compared with moderate continuous training. This intensity-based effect was observed despite both exercise intensities raising whole-body insulin sensitivity. This later finding of discordance with brain and periphery insulin action following high intensity exercise on tissue-specific glucose uptake, is consistent with the observation that people with increased brain glucose uptake in response to insulin have decreased insulin-stimulated skeletal muscle glucose disposal (108). Because exercise is known to increase skeletal muscle insulin sensitivity, it is paramount to understand the role exercise dose on affecting insulin-mediated brain glucose metabolism. Recently, wheel running in obese rats with T2D indicated that exercise was capable of improving insulin-stimulated posterior cerebral artery vasodilation in association with nitric oxide and reduced ET-1 signaling (109). Moreover, Ruegsegger et al. reported that exercise improved brain insulin sensitivity of rodents fed a high-fat diet (110). The mechanism by which exercise increased brain insulin sensitivity appears related to increased ATP and reduced ROS generation by mitochondria. Additional work is warranted to understand this brain-skeletal muscle “cross-talk” in order to better understand glycemic control responses to exercise.

Metformin has been suggested as a potential treatment for cognitive impairment (111). Because metformin has been shown to promote peripheral insulin sensitivity, it would be reasonable to expect an impact on the brain. A recent pilot trial was conducted whereby metformin was administered in patients with Alzheimer's disease (112). It was reported that metformin was linked to improved learning, memory, and attention in individuals with mild cognitive impairment. The reason metformin may improve this cognitive function in humans remains to be elucidated, but work in high-fat-fed rodents suggests that increased brain insulin sensitivity, as well as cerebral and hippocampal mitochondrial function, may play a role (113). In addition, metformin is capable of crossing the blood-brain barrier and regulating tau phosphorylation in mouse models, thereby minimizing risk for Alzheimer's disease (114).

To date, no studies have examined how metformin in combination with exercise affects brain regulation of glycemic control. This may be important given the collective body of literature demonstrates that metformin attenuates skeletal muscle insulin sensitivity (23, 32, 54), and skeletal muscle is a key tissue proposed to secrete myokines that affect brain function and cognition (115). Further work in this area is warranted to provide an improved understanding of how exercise and/or metformin benefit not only glycemic control but also reduce T2D and dementia risk in aging adults.

## Cellular Mechanism by Which Metformin Impacts Exercise Adaptation

Most agree that exercise or metformin therapy alone confer favorable effects on cellular pathways that regulate glycemic control across tissues for T2D and CVD risk reduction. The major concern at hand is the notion that 1 + 1 = 2 when considering exercise and metformin for cardiometabolic health. It now appears clear that the mechanism(s) by which exercise and metformin act to affect health interact on some yet to be determined pathway(s) that influences adaptation.

Aerobic fitness (i.e., VO_2_peak) is related to reduced risk for developing T2D independent of age and family diabetes history (76). Not surprisingly, elevations in VO_2_peak have been implicated in metabolic adaptations (e.g., mitochondrial biogenesis, oxidative enzymes) that are strongly associated with elevated insulin sensitivity (91). A reason metformin could constrain gains in aerobic fitness relates to the observation that metformin partially inhibits Complex 1 of the mitochondrial electron transport system (116). In turn, we examined the impact metformin has on VO_2_peak 10 weeks of exercise training in individuals with prediabetes (69). Exercise training alone significantly enhanced VO_2_peak by nearly 20%, while metformin plus exercise only increased by ~10%. This attenuated aerobic fitness adaptation has public health relevance since the combined treatment resulted in people exercising at a higher percentage of their post-training VO_2_peak of roughly 5% and consequently, people reported a higher perception of effort (via the Borg Scale) (69). This observation is consistent with new work highlighting that even acute administration of metformin raised perceptions of effort during exercise (117). Interestingly, new work highlights in older adults that 12 weeks of metformin treatment blunted the improvement in aerobic fitness by ~50% (33), which is consistent with our work in middle-aged adults with prediabetes (32). The implication of these findings is important as an increased perception of effort could lead to possibly a decrease in either long-term exercise adherence and/or changes in non-exercise physical activity behavior, thereby independently or collectively negatively influencing cardiometabolic health. However, it is worth acknowledging that not all studies confirm that metformin decreases VO_2_peak. In fact, some have shown metformin to raise exercise tolerance in people with coronary artery disease (118).

A possible reason metformin interacts with exercise-mediated skeletal muscle adaptation relates to lowering mitochondrial ROS generation (119). We previously hypothesized that skeletal muscle contraction induced ROS generation is an important mediator of glucose and insulin metabolism adaptation, in part based on literature showing anti-oxidants blunt exercise health benefit (120). Newer literature supports this idea suggesting that blunting NADPH oxidase 2 (NOX2)-mediated ROS, which is responsible for GLUT-4 translocation, blunts glucose uptake during muscle contraction in both human and mouse models (121). But, because metformin counters ROS signaling (119) in muscle, it is possible that the post-exercise cellular signals important for mitochondrial capacity (e.g., PGC-1a), blood flow (e.g., nitric oxide mediated endothelial function), glucose uptake (GLUT-4 translocation), as well as brain glucose metabolism that contribute to multi-organ insulin sensitivity, are blunted. This hypothesis was somewhat supported by prior work, whereby Sharoff et al. showed that metformin blunted the rise in AMPK activity during the immediate post-exercise period in insulin resistant adults, and this skeletal muscle observation directly correlated with attenuated insulin sensitivity (23). However, new work suggests that acute metformin treatment for 4 days did not affect AMPK activity during exercise in skeletal muscle or adipose tissue of lean healthy men. However, a novel observation was that metformin concentrations were detected in skeletal muscle, and it was proposed that longer duration (e.g., 5 days vs. 12 weeks) may be needed to elicit change in AMPK and/or mitochondrial content (117). We recognize though that not all studies support the action of metformin to reduce complex I of the mitochondria and impact indirectly AMPK, and this is an area of much debate (122). Indeed, recent work highlights that metformin may impede both the malate-aspartate as well as the glycerol-phosphate shuttle, thereby together increasing the cytosolic NADH:NAD+ ratio and allosterically inhibiting energetic processes that would support tissue function (49). Interestingly, it was proposed that metformin may impact immune function in older adults following resistance training, and alleviate inflammatory mediated processes that may hinder muscle accretion in response to resistance exercise (34). This is consistent with the notion that metformin promotes polarization from M1 pro-inflammatory macrophages to M2 anti-inflammatory macrophages (49) as well as induces autophagy to attenuate Th2 immune cell activation and inflammation (123). However, the results of the recent MASTERS trial showed no effect of metformin on resistance training-induced inflammation in skeletal muscle, despite the observation that lean body mass gains were blunted in relation to strength following the combined therapy compared with resistance exercise training alone. This was shown to parallel AMPK activation as well as inhibition of p70S6K1 phosphorylation (an immediate target of mTOR) (34). An additional or alternative explanation for the blunted muscle accretion post-training in the latter study may result from newer work showing that metformin reduces skeletal muscle autophagy and/or cell proliferation in C2C12 myotubes (124, 125), although data in humans following exercise training is unknown. Taken together, with possible influences of gastrointestinal adaptations with metformin of gut microbiota, bile acids, and/or GLP-1 (65), additional work is required to understand the exact cellular mechanisms by which metformin interacts with exercise across tissues for optimization of glycemic control. In fact, it is important to acknowledge that there are no suggestions for altered fasting glucose or liver insulin action in response to exercise plus metformin. Moreover, although elevated FFA levels have been detected following the combined therapy, no studies have been specifically designed to understand adipose insulin sensitivity following exercise plus metformin treatment. Nor has there been work examining the interaction of exercise and metformin on vasculature or brain insulin sensitivity to understand the importance of blood delivery and neural control of glucose metabolism. At this time, skeletal muscle appears to be a primary tissue regulating blood glucose, and additional cellular work is warranted to understand if these combined therapies lead to over-taxation of bioenergetic pathways that result in mal-adaptation. This may be particularly important since new work suggests that exercise may alter the pharmacokinetics and increase the bio-availability of metformin in circulation (126).

## Clinical Considerations and Conclusions

Developing precise exercise programs for maximal glycemic control remains to be identified. The collective literature suggests that, if anything, metformin attenuates the effects of exercise at improving insulin sensitivity at the level of skeletal muscle. Moreover, alterations in blood glucose, hypertension as well as inflammation have been noted. While no study to date has shown blood glucose to worsen as reflected by higher blood glucose concentrations relative to the start of the combined treatment, the literature highlights that there are either null, additive, or blunted effects on glycemia. The reason for this variability is not entirely clear but may relate to studies whereby people are habitual vs. naive metformin users or the outcome of interest. In either case, it is clear the magnitude of benefit will vary based on what tissue or outcome is of interest. Systemic studies determining the benefit of different exercise doses as well as risk factors of people (age, hypertension, dementia, T2D, etc.) co-prescribed metformin would enable individualized treatments that favor glycemic control. For instance, to date a basic biologic question is whether men or women respond differently to exercise plus metformin therapy based on underlying differences in aerobic fitness as well as muscle mass/fiber composition. Further, these gains in aerobic fitness and muscle mass are not only relevant to aging men and women with or without chronic disease, but also children and adolescents. It is well accepted that peak fitness/bone/muscle occur near the 3rd decade of life. But the effect of prescribing metformin with exercise in children and adolescents have on the rate of gain in these fitness outcomes is largely unknown in boys and girls. With emerging literature suggesting that off label or prophylactic use of metformin may be effective for weight management and obesity prevention in adolescents (54, 71, 127) more children may be provided metformin and recommended to exercise. This raises potential concern toward altered maturation growth rates and cardiometabolic risk during youth as well as then for later in life health risk compared with youth advised to exercise only with proper nutrition (54, 71). Thus, health care providers should be aware of these potential interactions to strike balance between current disease risk with long-term well-being. We also recognize that people are not often prescribed only one medication, and further work is warranted to tease out the effects of multiple pharmacological agents or even dietary supplements (e.g., metformin with GLP-1 agonists, SGLT-2 inhibitors, statins, antioxidants, etc.) in combination with exercise to gain a better understanding on glucose metabolism. However, it is important to acknowledge that recent work has suggested that other glycemic medications, including GLP-1 agonists and SGLT-2 inhibitors, have been shown to interact with exercise (128–130). This highlights the potential for medications to interfere or add with exercise-mediated glycemic benefit. Thus, there is potential for people to be at risk for developing T2D or cardiovascular abnormalities when co-prescribed treatments compared with those treated with exercise alone over time. Large-randomized clinical trials are critically needed to determine the effects combining exercise, with or without diet, and medications for improved evidenced-based practice.

## Author Contributions

SM wrote the majority of the review with NS providing edits. SM and NS collaborated on writing on the metformin and exercise on brain insulin sensitivity section. NS drafted the figure with SM providing edits.

## Conflict of Interest

The authors declare that the research was conducted in the absence of any commercial or financial relationships that could be construed as a potential conflict of interest.
